# The roles of lumbar load thresholds in cumulative lifting exposure to predict disk protrusion in an Asian population

**DOI:** 10.1186/s12891-020-3167-y

**Published:** 2020-03-16

**Authors:** Isabella Y.-J. Hung, Tiffany T.-F. Shih, Bang-Bin Chen, Saou-Hsing Liou, Ing-Kang Ho, Yue Leon Guo

**Affiliations:** 1 Department of Nursing, Chung Hwa University of Medical Technology, Tainan, Taiwan; 2 Department of Medical Imaging and Radiology, National Taiwan University Hospital and National Taiwan University (NTU), College of Medicine, Taipei, Taiwan; 3 National Institute of Environmental Health Sciences, National Health Research Institute (NHRI), Miaoli, Taiwan; 4 Center for Drug Abuse and Addiction, China Medical University Hospital, Taichung, Taiwan; 5 Graduate Institute of Clinical Medical Science, China Medical University, Taichung, Taiwan; 6 Department of Environmental and Occupational Medicine, College of Medicine, National Taiwan University (NTU) and NTU Hospital, Taipei, Taiwan; 7 Graduate Institute of Environmental and Occupational Health Sciences, College of Public Health, National Taiwan University (NTU), Taipei, Taiwan

**Keywords:** Cumulative, Lifting load, Cross-sectional study, Threshold, Disk protrusion

## Abstract

**Background:**

The purpose of this study was to determine whether a specific threshold per lifting movement, the accumulation above which best predicts lumbar disk protrusion, exists or the total lifting load should be considered.

**Methods:**

This was a retrospective study. Subjects with various lifting exposures were recruited. Disk protrusion was assessed by magnetic resonance imaging. The cumulative lifting load was defined as the sum of the time-weighed lumbar load for each job and was calculated using a biomechanical software system. The effectiveness of accumulation above different thresholds in predicting disk protrusion were compared using four statistical methods.

**Results:**

A total of 252 men and 301 women were included in the final analysis. For the men, 3000 Newtons for each lifting task was the optimal threshold for predicting L4-S1 disk protrusion, whereas for the women, 2800 Newtons was optimal.

**Conclusions:**

Our findings suggested that for cumulative lifting exposure, including the total lifting load without defining a minimal exposure limit might not be the optimal method for predicting disk protrusion. The NIOSH 3400 Newton recommended limits do not appear to be the optimal thresholds for preventing disk protrusion. Different lifting thresholds might be needed for men and women in the workplace for their safety.

## Background

Numerous studies have reported a relationship between occupation workload and disk protrusion [1–8], which may lead to sciatica, low back pain (LBP), and even long-term disability. Disk protrusion has been listed as an occupational disease and a compensatory condition in many countries, such as Denmark, France, Germany, the United States, and Taiwan [7]. In recent decades, researchers have reported that the cumulative lifting load is related to disk protrusion in a dose-dependent manner [5–8]. However, lifting objects with various weights is inevitable in everyday work and life. A crucial question is whether a specific threshold, the accumulation above which best predicts lumbar disk protrusion, exists or the total lifting load should be accumulated.

A review of the literature revealed several recommended lifting thresholds, although most of them were used in the prevention of low back injury [9–16]. For example, the National Institute for Occupational Safety and Health (NIOSH) of the United States suggested that if spinal compression exceeds approximately 3400 N (Newtons), workers are at an increased risk of low back injury [17]. Nevertheless, these recommended lifting limits might not be practical for calculating the cumulative effects for several reasons. First, they were determined for a single spontaneous lifting task, and the career-long effects of repeated lifting were not considered. Second, most of them were proposed for preventing LBP not disk protrusion. Third, the safe manual lifting threshold of 3400 N recommended by NIOSH does not appear to be optimal, as currently, more than 50% of work-related low back injuries are attributed to manual lifting tasks [18]. Fourth, uniform lifting limits are not generalizable across individuals of different ethnicities and sexes. Accordingly, this study was conducted to determine the optimal lifting threshold per lift by calculating the cumulative load to prevent disk protrusion in Asia and to determine whether the threshold differs between men and women.

## Methods

### Study population

This was a retrospective study. The protocol and consent forms of the study were reviewed and approved by the National Taiwan University Hospital Research Ethics Committee (NTUH-REC No.:200805047R). The method of recruiting the participants, measurements of work exposure, and imaging studies of the participants’ lumbar spines are detailed elsewhere [8]. To obtain a broad spectrum of lifting exposures, the following number of participants from 2 populations were recruited: (1) 263 walk-in clinic patients and (2) 452 workers who carry heavy loads. The patients visited the Internal Medicine Clinic of National Taiwan University Hospital, were diagnosed with upper respiratory infections (URIs), which were mostly the common cold, and were recruited as the initial study population. The group that carried heavy loads included workers from one fruit and vegetable wholesale market. Lifting is a daily, routine task for these workers. During recruitment, the market workers and the walk-in patients were not informed of the hypothesis of the study. They were invited to participate in an investigation regarding spine and bone disorders. The inclusion criteria were an age between 20 and 65 years and at least 6 months of working experience. Participants diagnosed with cancer, psychiatric conditions, spinal tumours, inflammatory spondylopathy, compression fractures, or major back trauma were excluded [8]. We combined the participants in the 2 populations to examine the effects of lifting on disk protrusion. Before participating in the study, all workers and patients received written and oral information regarding the study procedures and potential adverse effects and signed informed consent forms. Of these eligible 715 subjects, 162 were excluded from this study for the following reasons: 84 people experienced cancer, 27 people had major back trauma, 18 people had compression fractures, 16 people had psychiatric conditions, 13 people had spinal tumours, and 4 people had inflammatory spondylopathy.

### Data collection

Each participant was asked to complete a questionnaire and to undergo a magnetic resonance imaging (MRI) scan of the lumbar spine. A detailed structured interview was administered to the participants to assess the relevant work tasks in each job held since they entered the workforce. The occupational history included the participant’s job titles, tenures, body weights at each job, descriptions of the tasks, loads carried, lifting frequencies and durations, working hours per day and working days per week. The participants were encouraged to recall their body weights during the time they worked at each job. When the job period was longer than 5 years, the average body weight during this job period was used.

### Estimation of the lumbar disk compression load

A method of estimating lumbar disk compression load has been published previously [8]. Regarding the estimation of lifting exposure, the participants recalled all of the jobs they had held after completing schooling and the weight carried during, frequency of, and duration of each task. The participants performed a typical material handling task to simulate the positions and weights encountered at each job. The lifting activity was divided into a sequence of static postures, including the initial lift-up, transferring, and unloading postures, and each posture was analysed. The initial position in the weight lifting task was defined as the lift-up posture, the final position was defined as the unloading posture, and the action of transferring the material while walking was defined as the transferring posture. Although the initial and final positions of lifting may have varied during a typical day of materials handling on the job, the selected typical tasks, including the simulated positions and weights, were used to calculate the compression load experienced during the job. The compression load on the disk during lifting was estimated using the 3D Static Strength Prediction Program (3D SSPP, University of Michigan, Michigan) [19, 20]. Anthropometric data, such as the participant’s sex, height, body weight, and carried weight, and a photograph of each participant in the working posture were input into the system to predict the lumbar load. To evaluate the intrarater and interrater reliability of the lumbar load estimated by 3D SSPP, photographs of the simulated work conditions of the 60 study participants were repeatedly evaluated in 2 rounds, with the second round of evaluation conducted 4 weeks after the first round.

### Definition of the threshold in this study

The threshold in this study was defined as the lumbar load per lifting movement, and loads above this proposed value were considered to contribute to disk protrusion over an entire career and were included in the cumulative lifting exposure calculation. In other words, the calculation neglected all exposures with a lumbar load below the threshold. The proposed threshold values were set at zero Newtons (N) and all 100 N increments from 2000 to 4000 N.

### Calculation of the cumulative lifting exposure on the lumbar disk

The method of calculating the cumulative lifting exposure on the lumbar disk was modified from the Mainz-Dortmund dose model (MDD), which is based on overproportional weighting of the lumbar disc compression force relative to the corresponding duration of lifting, and has been applied in several studies [5–8].

To investigate the actual cumulative lifting exposure, the participants recalled details regarding the lift-up time (t_lift-up_), transporting time (t_transporting_), and unloading time (t_unload_) of each lifting task at their jobs. Hence, in this study, the lifting exposure of each task was defined as the sum of the product of the lift-up lumbar load (F_lift-up_) and the corresponding lift-up time, the product of the transporting lumbar load (F_transporting_) and the corresponding transporting time, and the product of the unloading lumbar load (F_unload_) and the corresponding unloading time. Only the lumbar loads larger than the proposed threshold were eligible for inclusion in the cumulative exposure calculation. For example, if the threshold is set at zero N, every load lifted in each activity is included in the calculation. When the threshold is set to be 3400 N, only exposures with a lumbar load per lift above 3400 N are eligible for inclusion in the calculation. For each job described, the lifting exposure was calculated as the sum of the product of the lifting load and the corresponding duration of lifting in hours (Newtons × hours, Nh). The cumulative lifting exposures for each participant were then calculated by summing the lifting exposures on the lumbar disk across all jobs.

The calculation can be expressed as the following equation:

$$ \mathrm{Cumulative}\ {\mathrm{lifting}\ \mathrm{exposure}}_{\left(\mathrm{Newtons}\times \mathrm{hours}\right)=\Sigma}\ \left[\left(\mathrm{F}\ \mathrm{lift}-{\mathrm{up}}_{\left(\mathrm{Newtons}\right)}\times \mathrm{t}\ \mathrm{lift}-{\mathrm{up}}_{\left(\mathrm{seconds}\right)}+\mathrm{F}\ {\mathrm{transporting}}_{\left(\mathrm{Newtons}\right)}\times \mathrm{t}\ {\mathrm{transporting}}_{\left(\mathrm{seconds}\right)}+\mathrm{F}\ {\mathrm{unload}}_{\left(\mathrm{Newtons}\right)}\times \mathrm{t}\ {\mathrm{unload}}_{\left(\mathrm{seconds}\right)}\right)\times {1}_{\left(\mathrm{minute}\right)}/{60}_{\left(\mathrm{seconds}\right)}\times {1}_{\left(\mathrm{hour}\right)}/{60}_{\left(\mathrm{seconds}\right)}\times \mathrm{frequency}\ \mathrm{of}\ \mathrm{lifting}/\mathrm{day}\times \mathrm{working}\ \mathrm{days}/\mathrm{year}\times \mathrm{working}\ \mathrm{year}\right] $$

where F represents the lifting load (Newtons) on the lumbar disk and t represents the duration of lifting the load (seconds).

An example of the calculation of the sum of the lifting exposures is given below:

One male worker with a body height of 170 cm and weight of 75 kg reported that he had carried object 1 (weighing 10 kg; lifted it up for 1 s, walked with it for 30 s, and unloaded for 1 s) 100 times a day and object 2 (weighting 20 kg; lifted it up for 1.5 s, walked with it for 10 s, and unloaded for 1.5 s) 50 times a day, for the 220 days a year that he worked. The total work tenure was 20 years.

The lumbar force estimated by 3D SSPP

**Table:** 

		Estimated lumbar force (Newtons)			Duration of one task (seconds)		
	weight	lift-up	walking	unload	lift-up	walking	unload
Object 1	10 kg	3713	1667	3713	1	30	1
Object 2	20 kg	4858	2343	4858	1.5	10	1.5

Calculation of the cumulative lifting exposure:

1. When the threshold is set at zero N, the cumulative exposure is as follows:

$$ \left\{\left[\left(3713\times 1\right)+\left(1667\times 30\right)+\left(3713\times 1\right)\right]\times 100+\left[\left(4858\times 1.5\right)+\left(2343\times 10\right)+\left(4858\times 1.5\right)\right]\times 50\right\}\times 220\times 20/3600=9.3\times {10}^6\ \left(\mathrm{Newton}-\mathrm{hours}\right) $$

The result of the calculation indicated that this person should be included in the intermediate lifting group (1.8 × 10^6^~1.6 × 10^7^ Newton-hours).

(2) When the threshold is set at 3000 N, the cumulative exposure is as follows:

$$ \left\{\left[\left(3713\times 1\right)+\left(3713\times 1\right)\right]\times 100+\left[\left(4858\times 1.5\right)+\left(4858\times 1.5\right)\right]\times 50\right\}\times 220\times 20/3600=1.79\times {10}^6\ \left(\mathrm{Newton}-\mathrm{hours}\right) $$

The result of the calculation indicated that this person should be included in the intermediate lifting group (2.5 × 10^5^~5.6 × 10^6^ Newton-hours).

(3) When the threshold is set at 3400 N, the cumulative exposure is as follows:

$$ \left\{\left[\left(3713\times 1\right)+\left(3713\times 1\right)\right]\times 100+\left[\left(4858\times 1.5\right)+\left(4858\times 1.5\right)\right]\times 50\right\}\times 220\times 20/3600=1.79\times {10}^6\ \left(\mathrm{Newton}-\mathrm{hours}\right) $$

The result of the calculation indicated that this person should be included in the intermediate lifting group (0~4 × 10^6^ Newton-hours).

(4) When the threshold is set at 4000 N, the cumulative exposure is as follows:

$$ \left\{\left[\left(4858\times 1.5\right)+\left(4858\times 1.5\right)\right]\times 50\ \right\}\times 220\times 20/3600=8.9\times {10}^5\ \left(\mathrm{Newton}-\mathrm{hours}\right) $$

The result of the calculation indicated that this person should be included in the intermediate lifting group (0~4 × 10^6^ Newton-hours).

We divided the participants into low, intermediate and high cumulative lifting load groups with even distributions of males and females according to the cumulative lifting exposure categories. However, only the lift-up forces larger than the proposed threshold were included in the calculation. Thus, as the threshold increased, more participants were categorized in the low lifting group. Therefore, males with a cumulative exposure above the 3000 N and zero N thresholds were categorized into the low, intermediate, and high tertiles. For those with a cumulative exposure above the 3400 N and 4000 N thresholds, the low group included the participants with zero Nh, and the remaining males with cumulative loads above zero Nh were split between the intermediate and high groups. On the other hand, in the female population, those with a cumulative exposure above the zero N threshold were divided into low, intermediate, and high tertiles. For those with a cumulative exposure above the 2800 N, 3400 N, and 4000 N thresholds, the low group included those with zero Nh, and the remaining females with cumulative loads above zero Nh were divided between the intermediate and high groups.

The reproducibility of the lifting measurements was tested 6 months after the initial interview with 25 participants. Their current jobs were used for reliability testing. These measurements included the working tenure, lifting weights, frequency of lifting per day, and lift-up time for the job. After observing and recording the fruit workers’ practices, we found that most of the participants’ lift-up times were nearly equal to their unloading times and that the transporting times were zero. Therefore, the reliability of the transporting time and unloading time were not examined. In addition, we determined that pushing or pulling is not a common task for the majority of fruit market workers because they typically drive an electric pedicab to transfer fruit boxes. Therefore, the lumbar load of pushing and pulling was not assessed.

Each intervertebral disk at L4–L5 to L5–S1 was evaluated for disk bulging, protrusion, extrusion, and sequestration using MRI. All MRI examinations were conducted at the National Taiwan University Hospital. The MRI equipment and protocol, definitions of the disk conditions, and evaluation of intrarater reliability regarding the presence or absence of protrusion are described in a previous study [8]. Two radiologists were responsible for the image interpretation and were blinded to the lifting exposure status of the participants.

### Data analysis

The reproducibility of the calculation of the lifting load and lifting measurements was analysed using SPSS version 16.0 for Windows (SPSS Inc., Chicago, Illinois), and intraclass correlation coefficients (ICCs) were computed. Kappa was used to assess the intrarater reliability of disk protrusion on MRI. Logistic regression analysis using JMP 5.0 (SAS Institute Inc., Cary, North Carolina) was used to identify the association between the cumulative lifting load and disk protrusion at either of the lower disk levels, namely, the L4-L5 and L5-S1 disks. Each variable was examined to determine its influence on disk protrusion and was considered to be a potential confounder if there was a statistically significant association (*p* < 0.05). Thus, the logistic regression was adjusted for the participant’s age, BMI, and history of smoking. Odds ratios (ORs) and 95% confidence intervals were calculated by logistic regression analysis. To determine the best threshold of the lifting load, four statistical values were used to compare the outcome (L4-S1disk protrusion) and the cumulative lifting load with different thresholds, namely, (1) the area under the curve (AUC) of a receiver operating characteristic (ROC) curve, (2) coefficient of determination (*R*^2^), (3) Akaike information criterion (AIC), and (4) Bayesian information criterion (BIC). We compared the AUC in various models that were plotted using MedCalc for Windows Version 9.2.1.0 (MedCalc Software, Mariakerke, Belgium). Models with higher AUC values were considered the optimal models. The amount of the cumulative lifting load variable that was explained by various threshold values in the model was evaluated based on the *R*^2^ statistic. The AIC and BIC were obtained using SAS Version 9.1 (SAS Institute Inc.). The AIC is closely related to the BIC. Given a set of candidate models for the data, the preferred model is the one with the smallest AIC value, and the same concept applies to the BIC.

## Results

A total of 553 volunteers were included in the final analysis; 252 participants were men (mean age 49.8 years, standard deviation (SD): 11.7), and 301 were women (mean age 51.3 years, SD: 9.4). The demographic characteristics of the participants are shown in Table 1. The men exhibited higher BMI values (25.6 + 3.1 kg/ m^2^) than the women did (24.1 + 3.8 kg/ m^2^), and most participants had more than 15 years of work experience (75.6%). LBP during the past 6 months was reported by approximately 83.6% of the participants. The reproducibility of the lifting measurements was high for the working tenure (ICC =0.943), lifted weight (ICC =0.945), and frequency of lifting per day (ICC =0.914), and it was moderate for the lift-up time (ICC =0.743). The intrarater and interrater reliability values of the lifted load calculation were 0.998 and 0.992 (ICC), respectively. The Kappa value of the intrarater reliability for L4-S1disk protrusion was 0.850, which was considered good.

**Table 1:** Demographic characteristics of the study participants

Variables	Male, ***N*** = 252 N (%)	Female, ***N*** = 301 N (%)	All, ***N*** = 553 N (%)
Age, mean + SD (years)	49.8 + 11.7	51.3 + 9.4	50.6 + 10.5
< 40	55 (21.8)	37 (12.3)	92 (16.6)
40~ < 50	51 (20.2)	71 (23.6)	122 (22.1)
50~ < 60	95 (37.7)	142 (47.2)	237 (42.9)
> 60	51 (20.2)	51 (16.9)	102 (18.4)
BMI, mean + SD (kg/m^2^)	25.6 + 3.1	24.1 + 3.8	24.8 + 3.5
< 24	73 (29.0)	151 (50.2)	224 (40.5)
24~ < 27	103 (40.8)	93 (30.9)	196 (35.4)
> 27	76 (30.2)	57 (18.9)	133 (24.1)
Lifetime work tenure (years)			
< 15	59 (23.4)	76 (25.2)	135 (24.4)
15~ < 30	82 (32.5)	127 (42.2)	209 (37.8)
> 30	111 (44.0)	97 (32.3)	208 (37.7)
missing	0 (0.0)	1 (0.3)	1 (0.1)
Low back pain (within 6 months)	211 (84.1)	246 (83.1)	457 (83.6)
Cigarette smoking (pack-years)			
0	138 (54.7)	288 (95.7)	426 (77.0)
1~ < 20	43 (17.1)	13 (4.3)	56 (10.1)
> 20	70 (27.9)	0 (0.1)	70 (12.7)
missing	1 (0.3)	0 (0.0)	1 (0.2)
Exercise^a^ (Yes)	171 (67.9)	185 (62.5)	356 (65.0)

*BMI* body mass index, *SD* standard deviation

^a^Yes means ever having regular exercise for 30 min or longer each session, at least one session per week, minimum duration of 3 months, from age of 12 years to the present time

**Fig. 1:**
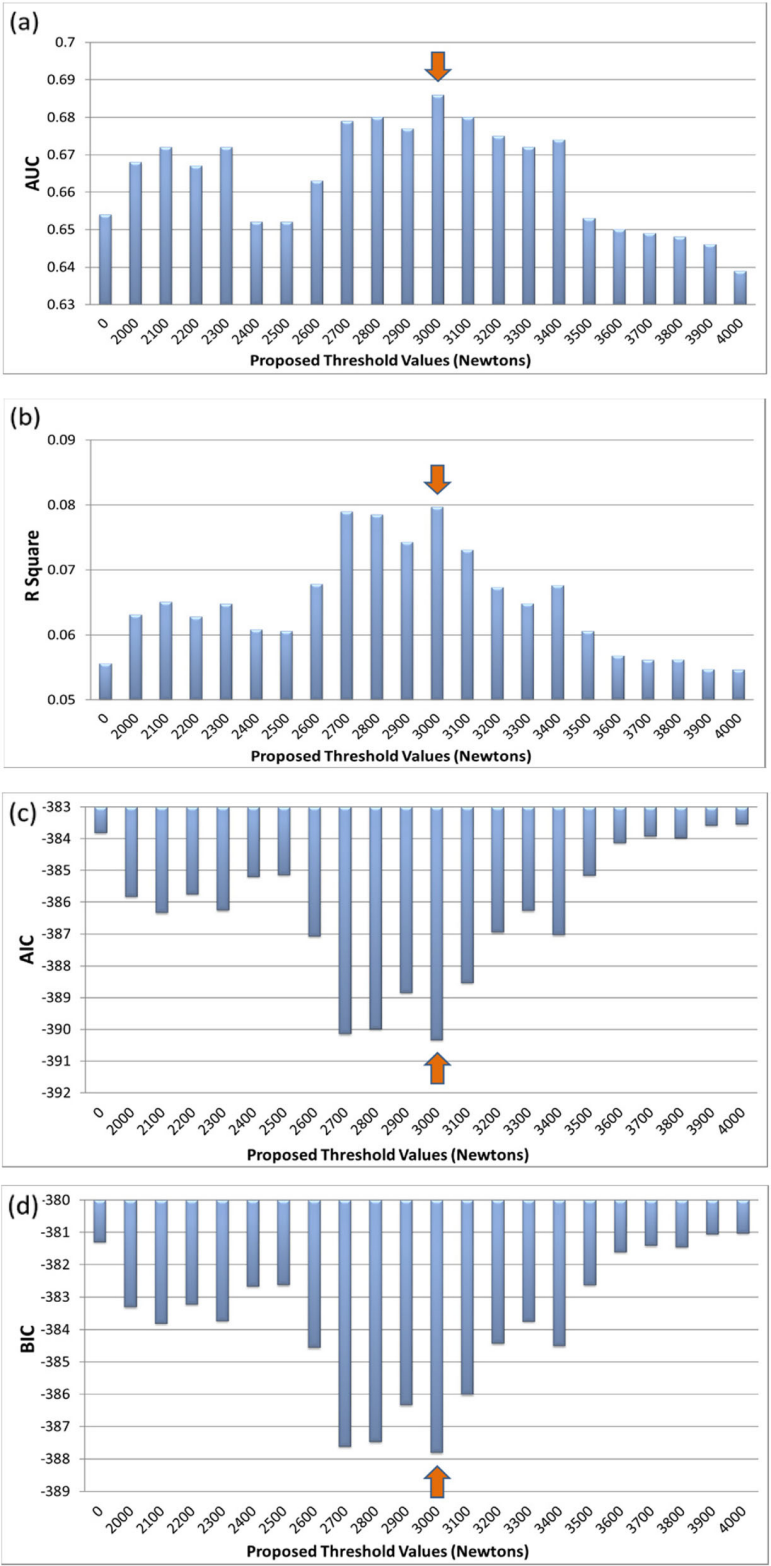
**a** The AUC statistic distrubution of L4-S1 disc protrusion with proposed threshold values in male participants. **b** The R Square values of L4-S1 disc protrusion with proposed threshold values in male participants. **c** The AIC values of L4-S1 disc protrusion with proposed threshold values in male participants. **d** The BIC values of L4-S1 disc protrusion with proposed threshold values in male participants

**Fig. 2:**
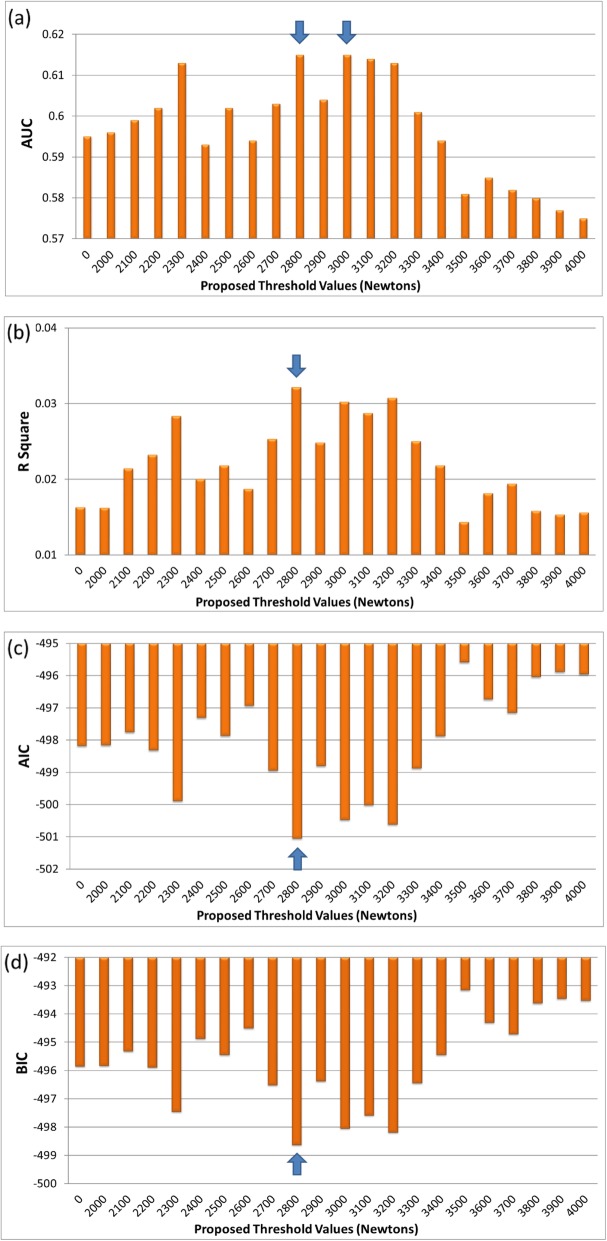
**a** The AUC statistic distrubution of L4-S1 disc protrusion with proposed threshold values in female participants. **b** The R Square values of L4-S1 disc protrusion with proposed threshold values in female participants. **c** The AIC values of L4-S1 disc protrusion with proposed threshold values in female participants. **d** The BIC values of L4-S1 disc protrusion with proposed threshold values in female participants

Figures 1 and 2 show the ability of the cumulative lifting load with different thresholds to predict L4-S1 disk protrusion in male and female participants, respectively.

**Fig. 3:**
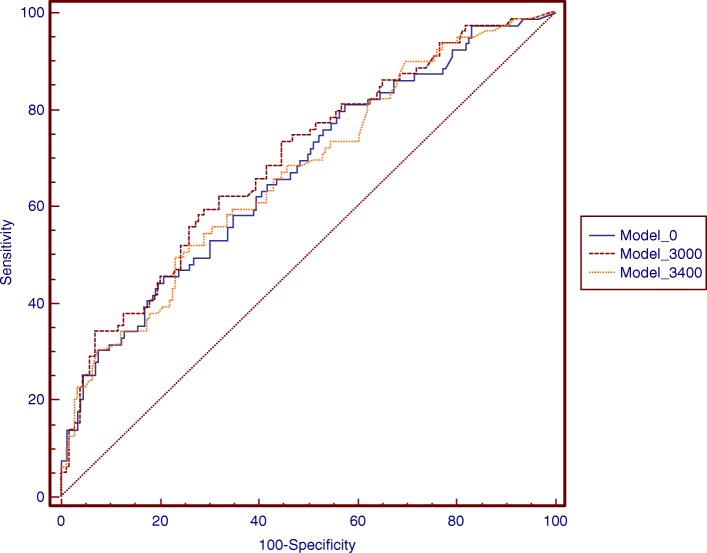
Receiver-operating characteristic curves for the prediction of L4-S1 disc protrusion in male participants by models of different threshold of lifting load. Model 0: AUC (95% CI) = 0.65 (0.61 - 0.71). *P* = 0.0001. Model 3000: AUC (95% CI) = 0.69 (0.63 - 0.74). *P* = 0.0001. Model 3400: AUC (95% CI) = 0.67 (0.61 - 0.73). *P* = 0.0001

**Fig. 4:**
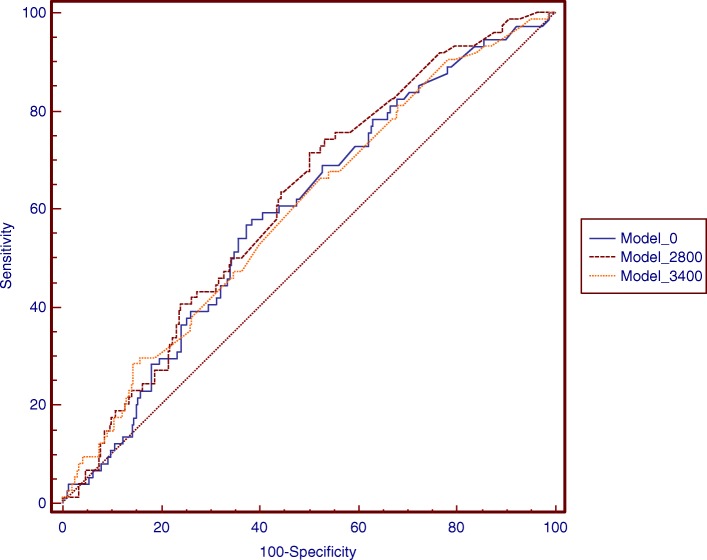
Receiver-operating characteristic curves for the prediction of L4-S1disc protrusion in female participants by models of different threshold of lifting load. Model 0: AUC (95% CI) = 0.60 (0.54 - 0.65). *P* = 0.0154. Model 2800: AUC (95% CI) = 0.62 (0.56 - 0.67). *P* = 0.0031. Model 3400: AUC (95% CI) = 0.59 (0.54 - 0.65). *P* = 0.0159

Detailed information is shown in Supplemental Table 1 and Supplemental Table 2. With any of the threshold values, the cumulative lifting load was significantly associated with L4-S1 disk protrusion. Among the male participants, the maximal AUC (0.686) was found when a lifting load of 3000 N was used as the threshold (Fig. 1a and Supplemental Table 1). The *R*^2^ statistic (0.0797), AIC (− 390.3), and BIC (− 387.8) were also optimal with the 3000 N threshold (Fig. 1b, c, d and Supplemental Table 1). The ROC curves of the 3400 N, 3000 N, and zero N models in the males are shown in Fig. 3. Among the female participants, the maximal AUC (0.615) was found when lifting loads of both 2800 N and 3000 N were used as thresholds (Fig. 2a and Supplemental Table 2). The *R*^2^ statistic (0.0321), AIC (− 501.1), and BIC (− 498.6) were also optimal when the 2800 N threshold was used (Fig. 2b, c, d and Supplemental Table 2). The ROC curves of the 3400 N, 2800 N, and zero N models in females are shown in Fig. 4.

Tables 2 and 3 show the adjusted odds ratios (aORs) for disk protrusion when the cumulative load was calculated with various thresholds included as predictors. In males, the cumulative load with the 3000 N and zero N thresholds was categorized into low, intermediate, and high tertiles. For the 4000 N and 3400 N thresholds, the low group was indicated (zero Nh), and there was a dichotomy (intermediate and high groups) among those with cumulative loads above zero Nh. A cumulative load of above 3000 N threshold had a stronger and significant association with L4-S1 disk protrusion (aOR =3.1, 95% confidence interval (CI) 1.5–6.7; aOR = 2.9, 95% CI 1.4–6.2) than did the loads above the zero N, 3400 N, and 4000 N thresholds (Table 2). In females, a cumulative load with a zero N threshold was categorized into low, intermediate, and high tertiles. For the 4000 N, 3400 N and 2800 N thresholds, the low group was indicated (zero Nh), and there was a dichotomy (intermediate and high) among those with cumulative loads above zero Nh. The cumulative load above 2800 N provided a stronger and significant association with L4-S1 disk protrusion (aOR =2.6, 95% CI 1.0–6.2; aOR =2.7, 95% CI 1.4–5.4; aOR =2.3, 95% CI 1.1–4.8) than did the loads above the zero N, 3400 N, and 4000 N thresholds (Table 3).

**Table 2:** The association between L4-S1 disk protrusion and lifetime cumulative lifting load in male participants

Lifetime cumulative lifting load (Newton-hr)			n	Disk protrusion at lower disk level (L4-S1)
				AOR
Only lift load above 4000 N was included	Low	0	137	1
	Intermediate	0~ < 4.0 × 10^6^	58	1.6 (0.8–3.1)
	High	> 4 × 10^6^	57	1.9 (1.0–3.8)
Only lift load above 3400 N was included	Low	0	96	1
	Intermediate	0~ < 4.0 × 10^6^	73	1.6 (0.8–3.3)
	High	> 4 × 10^6^	83	2.0* (1.0–3.9)
Only lift load above 3000 N was included	Low	< 2.5 × 10^5^	84	1
	Intermediate	2.5 × 10^5^ ~ < 5.6 × 10^6^	83	2.9*** (1.4–6.2)
	High	> 5.6 × 10^6^	85	3.1** (1.5–6.7)
lift load above 0 N was included	Low	< 1.8 × 10^6^	83	1
	Intermediate	1.8 × 10^6^~ < 1.6 × 10^7^	84	2.3* (1.2–4.9)
	High	> 1.6 × 10^7^	85	2.0 (1.0–4.2)

Adjusted for age, BMI, smoking

Statistically significant: *, *P* < .05; **, *P* < .01; ***, *P* < .001

**Table 3:** The association between L4-S1 disk protrusion and lifetime cumulative lifting load in female participants

Lifetime cumulative lifting load (Newton-hr)			n	Disk protrusion at lower disk level (L45-S1)
				AOR
Only lift load above 4000 N was included	Low	0	254	1
	Intermediate	0~ < 2.0 × 10^6^	24	0.7 (0.2–1.9)
	High	> 2.0 × 10^6^	23	2.6* (1.0–6.2)
Only lift load above 3400 N was included	Low	0	206	1
	Intermediate	0~ < 2.5 × 10^6^	47	1.6 (0.7–3.4)
	High	> 2.5 × 10^6^	48	1.9 (0.9–3.9)
Only lift load above 2800 N was included	Low	0	142	1
	Intermediate	0~ < 1.8 × 10^6^	79	1.9 (0.9–3.7)
	High	> 1.8 × 10^6^	80	2.7** (1.4–5.4)
lift load above 0 N was included	Low	< 1.26 × 10^5^	99	1
	Intermediate	1.26 × 10^5^ ~ < 5.6 × 10^6^	101	2.0 (1.0–4.1)
	High	> 5.6 × 10^6^	101	2.3* (1.1–4.8)

Adjusted for age, BMI, smoking

Statistically significant: *, *P* < .05; **, *P* < .01; ***, *P* < .001

## Discussion

In this study, optimal thresholds for the loads per lift that allow the best prediction of disk protrusion with the calculation of the cumulative exposure were found. The cumulative lifting load yielded the best prediction for L4-S1 disk protrusion when the threshold was set at 3000 N for male participants and 2800 N for female participants. Furthermore, the study was conducted in an Asian population, and the results might be more applicable in Asia than in other countries because uniform lifting limits might not be generalizable across individuals of different ethnicities and sexes.

One of the recommended lifting limits, the NIOSH 3400 N, is widely used by ergonomists as well as health and safety practitioners [12, 21]. It is based on the studies by Evans and Lisner [22] and Sonoda [23]. These studies show that microfractures of the vertebral cartilage endplates started to occur in the cadavers of subjects aged 60 or more years when average axial loads of 3400 N were applied. The major limitations of the NIOSH 3400 N recommendation are that the results are based on cadaver studies and on the immediate effects on the vertebral cartilage endplate rather than the cumulative effects. Our study is an important complement to the NIOSH 3400 N criteria and provides recommendations for long-term lifting limits.

To the best of our knowledge, only a few studies have examined the dose-effect relationship between the cumulative lifting load and disk protrusion. Seilder [5] conducted a thorough investigation of all past lifting loads for the participants. They showed that male workers who had been exposed to cumulative lifting loads of 5–21.5 × 10^6^ Nh exhibited a 1.7-fold higher risk of disk protrusion compared to those exposed to loads of 0 – < 5 × 10^6^ Nh, suggesting that cumulative effects of all lifting loads, without a threshold, on disk protrusion [5–7]. In a later study [8], participants who had been exposed to a high lifting load (≥8.9 × 10^6^ Nh) exhibited an OR of 2.2 for disk protrusion compared to those exposed to a low lifting load (< 4.9 × 10^5^ Nh). This last study also assumed no per lift threshold for the accumulated load. In this current study, the concept of a threshold per lift load was tested, and the results showed that applying certain thresholds provided better predictions in calculating the cumulative lifting load than not applying thresholds. Our findings suggested that when calculating cumulative lifting exposure, including the total lifting load without defining a minimal exposure limit might not be the optimal method for predicting disk protrusion. In addition, the investigation was carried out in an Asian population; therefore, the findings of this study might have limited generalizability to other populations.

Considering disk protrusion as the health outcome, the male participants seemed to tolerate a higher lumbar load than females on a per lift load basis. It is possible that the men generally had larger cross-sectional areas in the lower lumbar disks than the women [24]. The larger areas allowed men to endure a higher compression load. Thus, the results of this investigation suggest that different lifting thresholds should be applied to men and women in the workplace for their safety.

The strengths of our study include the detailed investigation of the cumulative lifting exposure, outcome assessment that was performed by using MRI, and application of the concept of a threshold per lift for cumulative lifting load calculations. However, there were several limitations in this study. First, the AUC, *R*^2^, AIC, and BIC statistics were the summary scores of prediction that were used for each threshold value. They did not allow for statistical comparisons among the proposed threshold values. Second, we relied on the participants’ memories regarding their occupational history and relevant work tasks performed several decades ago. Although the repeatability of the current job tasks was examined and found to be satisfactory, the reliability of the information pertaining to previous jobs was difficult to determine. To enhance reliability, a structured interview was executed to provide the participants with adequate time to recall the details related to the work they performed at their previous jobs. The trained interviewers used life milestones to help the participants recall the necessary details and took the working simulation photos by following a standard procedure. Previous studies have indicated that self-reported data does not provide satisfactory validity [25]. However, Pope assessed the accuracy of self-reported information on manual material handling activities and presented satisfactorily accurate results regarding the frequency, duration, and amplitude [26]. Direct measurements obtained using work or laboratory simulations yield the most accurate information; however, it is impractical to use such methods in retrospective studies involving relatively large sample sizes.

Another limitation is that we did not consider the loads lifted during leisure and home activities. In Kelsey’s study [27], the authors found that there was no association between acute herniation and participation in baseball, softball, jogging, golf, bowling, tennis, swimming or bicycling. However, another study [28] showed that leisure activities involving gardening, snow clearing, or building of a summer cottage were associated with more occurrences of disc degeneration in the upper lumbar levels, but it rarely snows and people rarely build summer cottages in Taiwan. Therefore, the risk factors for leisure and home activities were not thoroughly investigated in our study. This might have potentially caused misclassification and error in the cumulative lifting load estimates. The R^2^ values in males ranged from 0.0546 to 0.0797, and in females, it ranged from 0.0143 to 0.0321; these values reported in the study are very low. These results are consistent with Battie’s finding [28]. In the L4–5 and L5S1 regions assessed in this study, physical loading only explained 2% of the variance in disc degeneration. With the addition of age to the model, the explained variability in the discs increased from 2 to 9% and to 43% with the addition of familial aggregation. Thus, genetic factors greatly influence disc degeneration. This might be the reason that the explanatory power of lifting loading on disc protrusion was small.

Disk protrusion has been reported to be associated with manual handling activities [1–8], which may lead to low back pain. There are also many other potential risk factors for disk herniation. However, in Jensen’s study [29], the authors found that 64% of asymptomatic individuals had lumbar disk abnormalities (disk bulge, protrusion, or extrusion). Moreover, the cause of low back pain is also multifactorial, involving factors such as disk degeneration, disk herniation, ligament/tendon damage, muscle strains, manual handling activities, vibration, smoking, and psychological factors. For these reasons, we used lifting load as the only measurement to determine the development of disk protrusion, even though we adjusted for other potential risk factors, including the participant’s age, BMI, and smoking status. Although disk herniation is often seen in asymptomatic populations, setting a lifting threshold in the workplace is important to prevent workers from developing low back pain. It may be a useful tool for risk assessment and for prevention purposes to reduce the occurrence of injuries and medical costs.

In a previous study [8], Hung examined the associations between cumulative lifting load and lumbar disk degeneration and found a dose-response relationship. An additional question was raised regarding whether each lifting load was attributed to the outcome and should be accumulated. For example, a male worker with a body height of 170 cm and body weight of 75 kg carried a 2 kg object 5 times a day for 10 years. The compression force to the lumbar disk from this 2 kg object might not contribute to the development of disk protrusion, even though the worker carried it 5 times a day for 10 years. The cumulative calculation approach by summing all lifting loads can lead to overestimations. For this reason, we assumed that each disk condition might have the corresponding threshold. The study hypothesis is that there is a specific threshold for each lifting load, and cumulative exposure with loads above this threshold can lead to disk protrusion. Based on this hypothesis, this study attempted to investigate various lifting load values to determine the optimal threshold for the prediction of disk protrusion. Consequently, lifting loads below the threshold were neglected in the cumulative calculation, and in some circumstances, the final cumulative lifting load led to zero Nh as the cumulative exposure. Thus, these participants with zero Nh were finally included in the low lifting exposure group, even though these participants did not actually experience zero Nh at work. In the establishment of the thresholds, this approach was used to eliminate some extent of cumulative loading, but it may cause underestimations when the results are interpreted. The method using cumulative calculation with a threshold does not represent the entire cumulative exposure experienced by the participants. Therefore, a limitation of this study is that the threshold analysis performed could not be considered an accurate cumulative loading assessment according to our established techniques.

To determine the cumulative lifting exposure, we investigated the lumbar compression force the workers were exposed to (force), time of exposure (duration), and repetitive task exposure (frequency). The cumulative lifting exposure calculation method utilized in this study, which involved multiplying the force in Newtons by the duration has been used in previous studies [5–8]. One question that was raised when multiplying the force in Newtons by time was whether the injury accrued by a short duration with a high load might be equivalent to that accured by a longer duration with low load. However, it appears to be true that exposure to large loads causes more severe damage to tissues than small loads. That is, one reason we utilized the threshold in the multiplying the force in Newtons by time was to assess the cases with long durations and small loads. It is a limitation that the approach may not be the best method of assessing the effects of cumulative loading for musculoskeletal tissues. When exposed to repetitive stresses, musculoskeletal tissues experienced fatigue failure during cumulative damage development. In Gallagher’s study [30], the authors suggested that there are several established techniques for assessing the effects of cumulative loading using the fatigue failure theory. By using these techniques, a dose-response relationship between the estimated cumulative damage and musculoskeletal disorders was observed. Such relationships have been demonstrated for the low back, shoulder, and upper extremities. Additional research studies involving the principles of fatigue failure analysis in cumulative exposure assessments on disk degeneration need to be conducted.

## Conclusions

In this study, we applied the concept of a threshold per lift to the calculation of the cumulative lifting load. Our findings suggested that considering the total lifting load in the exposure calculation without defining a minimal exposure limit might not be a practical approach for predicting disk protrusion. In addition, the NIOSH 3400 N recommendation may not be the optimal threshold for preventing disk protrusion. Different lifting thresholds might be applied to men and women in the workplace to prevent injury.

## Supplementary information

**Additional file 1. ** Supplemental Table 1. Performance of predictive abilities for L4-S1 disk protrusion as measured by area-under-curve (AUC) of receiver-operator characteristic (ROC) curve, R-square, Akaike information criterion (AIC), and Bayesian information criterion (BIC) of cumulating lifetime lifting load using various threshold values in male participants. Supplemental Table 2. Performance of the predictive abilities for L4-S1 disk protrusion as measured by AUC of ROC curve, R-square, AIC, and BIC of cumulating lifetime lifting load using various threshold values in female participants.

## Data Availability

The dataset used for analysis during the current study are available from the corresponding author on reasonable request.

## Abbreviations

3D SSPP: 3D Static Strength Prediction Program
AIC: Akaike information criterion
aORs: Adjusted odds ratios
AUC: Area under the curve
BIC: Bayesian information criterion
BMI: Body mass index
CI: Confidence interval
ICCs: Intraclass correlation coefficients
JMP: Statistic software, SAS Institute Inc., Cary, North Carolina
kg/ m^2^: Kilogram per square meter
L4: The fourth lumbar spine vertebra
L5: The fifth lumbar spine vertebra
MedCalc: MedCalc Software, Mariakerke, Belgium
MRI: Magnetic resonance imaging
N: Newton
Nh: Newton × hour
NIOSH: National Institute for Occupational Safety and Health
NTUH-REC: National Taiwan University Hospital Research Ethics Committee
*P*: *p* value
*R*^2^: Coefficient of determination
ROC: Receiver operating characteristic
S1: The first sacrum spine vertebra
SD: Standard deviation
SPSS: Statistic software, SPSS Inc., Chicago, Illinois
URI: Upper respiratory infections
