# Supplementary material for: The roles of lumbar load thresholds in cumulative lifting exposure to predict disk protrusion in an Asian population
Source: BMC Musculoskelet Disord. 2020 Mar 16;21:169. doi: 10.1186/s12891-020-3167-y (PMC7077115; doi:10.1186/s12891-020-3167-y)
Supplement: Supplementary file 1 — Additional file 1. Supplemental Table 1. Performance of predictive abilities for L4-S1 disk protrusion as measured by area-under-curve (AUC) of receiver-operator characteristic (ROC) curve, R-square, Akaike information criterion (AIC), and Bayesian information criterion (BIC) of cumulating lifetime lifting load using various threshold values in male participants. Supplemental Table 2. Performance of the predictive abilities for L4-S1 disk protrusion as measured by AUC of ROC curve, R-square, AIC, and BIC of cumulating lifetime lifting load using various threshold values in female participants. [file 12891_2020_3167_MOESM1_ESM.docx]

Supplemental Table 1.

Performance of predictive abilities for L4-S1 disk protrusion as measured by area-under-curve (AUC) of receiver-operator characteristic (ROC) curve, R-square, Akaike information criterion (AIC), and Bayesian information criterion (BIC) of cumulating lifetime lifting load using various threshold values in male participants

| **Proposed threshold value (Newton)** | **AUC of ROC** | **R-Square** | **AIC** | **BIC** |
| --- | --- | --- | --- | --- |
| 0 | 0.654 | 0.0556 | -383.820 | -381.297 |
| 2000 | 0.668 | 0.0631 | -385.829 | -383.306 |
| 2100 | 0.672 | 0.0650 | -386.332 | -383.809 |
| 2200 | 0.667 | 0.0628 | -385.749 | -383.227 |
| 2300 | 0.672 | 0.0647 | -386.257 | -383.734 |
| 2400 | 0.652 | 0.0608 | -385.196 | -382.673 |
| 2500 | 0.652 | 0.0606 | -385.145 | -382.623 |
| 2600 | 0.663 | 0.0678 | -387.078 | -384.555 |
| 2700 | 0.679 | 0.0790 | -390.142 | -387.620 |
| 2800 | 0.680 | 0.0785 | -389.994 | -387.471 |
| 2900 | 0.677 | 0.0743 | -388.850 | -386.328 |
| 3000 | 0.686 | 0.0797 | -390.331 | -387.809 |
| 3100 | 0.680 | 0.0731 | -388.531 | -386.009 |
| 3200 | 0.675 | 0.0673 | -386.951 | -384.429 |
| 3300 | 0.672 | 0.0648 | -386.269 | -383.747 |
| 3400 | 0.674 | 0.0676 | -387.029 | -384.506 |
| 3500 | 0.653 | 0.0606 | -385.154 | -382.631 |
| 3600 | 0.650 | 0.0568 | -384.134 | -381.611 |
| 3700 | 0.649 | 0.0561 | -383.930 | -381.407 |
| 3800 | 0.648 | 0.0562 | -383.976 | -381.454 |
| 3900 | 0.646 | 0.0547 | -383.577 | -381.054 |
| 4000 | 0.639 | 0.0546 | -383.546 | -381.023 |

The association between lifetime lifting load for L4-S1 disk protrusion were analyzed by using logistic regression, adjusting for age, body mass index (BMI), and smoking

Supplemental Table 2.

Performance of the predictive abilities for L4-S1 disk protrusion as measured by AUC of ROC curve, *r*^2^, AIC, and BIC of cumulating lifetime lifting load using various threshold values in female participants

| **Proposed threshold value (Newton)** | **AUC of ROC** | **R-Square** | **AIC** | **BIC** |
| --- | --- | --- | --- | --- |
| 0 | 0.595 | 0.0163 | -498.187 | -495.855 |
| 2000 | 0.596 | 0.0162 | -498.160 | -495.828 |
| 2100 | 0.599 | 0.0214 | -497.756 | -495.320 |
| 2200 | 0.602 | 0.0232 | -498.324 | -495.889 |
| 2300 | 0.613 | 0.0283 | -499.892 | -497.456 |
| 2400 | 0.593 | 0.0200 | -497.319 | -494.884 |
| 2500 | 0.602 | 0.0218 | -497.879 | -495.443 |
| 2600 | 0.594 | 0.0187 | -496.938 | -494.503 |
| 2700 | 0.603 | 0.0253 | -498.948 | -496.513 |
| 2800 | 0.615 | 0.0321 | -501.061 | -498.625 |
| 2900 | 0.604 | 0.0248 | -498.812 | -496.377 |
| 3000 | 0.615 | 0.0302 | -500.478 | -498.043 |
| 3100 | 0.614 | 0.0287 | -500.020 | -497.584 |
| 3200 | 0.613 | 0.0307 | -500.626 | -498.191 |
| 3300 | 0.601 | 0.0250 | -498.876 | -496.441 |
| 3400 | 0.594 | 0.0218 | -497.882 | -495.447 |
| 3500 | 0.581 | 0.0143 | -495.593 | -493.158 |
| 3600 | 0.585 | 0.0181 | -496.748 | -494.312 |
| 3700 | 0.582 | 0.0194 | -497.155 | -494.719 |
| 3800 | 0.580 | 0.0158 | -496.054 | -493.619 |
| 3900 | 0.577 | 0.0153 | -495.894 | -493.459 |
| 4000 | 0.575 | 0.0156 | -495.970 | -493.534 |

The association between lifetime lifting load for L4-S1 disk protrusion were analyzed by using logistic regression, adjusting for age, body mass index (BMI), and smoking
